# The influence of physical activity and yoga on central arterial stiffness

**DOI:** 10.1186/1476-5918-7-2

**Published:** 2008-01-28

**Authors:** Courtney M Duren, Marie E Cress, Kevin K McCully

**Affiliations:** 1 Department of Kinesiology, University of Georgia, 115 Ramsey Center, Athens, GA, 30602, USA

## Abstract

**Purpose:**

Central arterial stiffness is an accepted risk factor for cardiovascular disease. While aerobic activity is associated with reduced stiffness the influence of practicing yoga is unknown. The aims of this study were to: 1) evaluate arterial stiffness in middle-aged adults who regularly practiced yoga, performed regular exercise, or were inactive, 2) evaluate the reproducibility of arterial stiffness measured in the left and right carotid artery and by pulse wave velocity (PWV).

**Methods:**

Twenty six healthy subjects (male and female, 40–65 yrs old) were tested on two separate days. Carotid artery distensibility (DC) was measured with ultrasound. Physical activity was determined by questionnaire.

**Results:**

Yoga and aerobic subjects had similar physical activity levels. Yoga and aerobic groups were not different in either DC (p = 0.26) or PWV (p = 0.21). The sedentary group had lower DC and higher PWV compared to the aerobic and yoga groups (both, p < 0.001). Stiffness measures were reliable day to day (coefficients of variation ~2.5%) and similar between left and right arteries (CV = 2.2%).

**Conclusion:**

Physical activity was a strong predictor of both measures of arterial stiffness, although other factors such as nutritional status need to be accounted for. An independent effect of practicing yoga could not be detected. Stiffness measures were reproducible and left and right sides were consistent with each other.

## Introduction

Cardiovascular disease is a major cause of death in the United States. Arterial stiffness in the central arteries has been implicated in the progression of cardiovascular disease[1]. Central arterial stiffness also has been shown to increase with age, starting with 40–60 year old subjects.

Aerobic exercise has been shown to reduce the age-related increase in arterial stiffness[2]. While a number of studies have shown that practicing yoga reduces blood pressure [3-5], there are different styles of yoga that vary in terms of exercise intensity. Hatha yoga is a common style of yoga that involves the use of inversion postures[6,7]. If inversion postures increase blood pressure in the carotid arteries, it is possible that this would be a stimulus to reduce arterial compliance. Resistance exercise has been shown to acutely reduce arterial compliance [8], and chronic resistance training is associated with reduced arterial compliance [9]. Based on these results, is not clear whether practicing physically active forms of yoga, including those with inversion postures would influence arterial stiffness in middle-aged adults.

Previous studies have shown that measuring carotid arterial stiffness has been shown to be reproducible to within 10%[10]. However, ultrasound measurements are often performed on one carotid artery, and cardiovascular disease is known to be asymmetrical in its presentation[6,7]. PWV measurements have been performed using either ECG of the heart or carotid-femoral tonometry to time the pressure wave[2]. ECG measurements have the disadvantage of assuming a constant isovolumetric contraction time while tonometry measurements require a skilled operator.

The purpose of this study was to measure central artery stiffness in three groups of healthy middle-aged adults (sedentary, moderately aerobically active, or who performed yoga). Central artery stiffness was measured on two days with ultrasound of the left and right common carotid arteries, and with PWV (ECG and carotid-femoral tonometry). We hypothesized that the regular practice of yoga with inversion postures would be associated with reduced arterial stiffness, that the measures of arterial stiffness would be reproducible, and that there would be a good agreement between the different measurements of central arterial stiffness.

## Methods

### Subjects

A total of 26 healthy men and women 40–64 years of age were studied. Sedentary subjects performed one or fewer bouts of vigorous aerobic activity a week in the previous year (N = 8); Yoga subjects performed yoga, including inversion positions at least 2 days a week in the previous year (N = 8) and aerobic subjects performed some form of aerobic exercise (walking, cycling, aerobics) three or more days a week for at least 30 minutes a day over the last year (N = 10). The Baecke Habitual Physical Activity Questionnaire was used to quantify physical activity levels[11].

All subjects were normotensive (<140/90 mmHg), nonobese, and free of chronic diseases by self-report. Subjects who used vasoactive, hypertensive, diabetic, or any other medicines or supplements that directly or indirectly affect cardiovascular function were excluded from the study. All subjects gave their written informed consent to participate. All procedures were reviewed and approved by the Internal Review Board at the University of Georgia.

### Testing procedures

Participants abstained from caffeine and any form of exercise for at least 2 hours before testing. All participants but one (N = 26) were tested on two separate days at approximately the same time of day. On the first visit the subjects filled out the Baecke Questionnaire of Habitual Physical Activity and answered questions on yoga and nutrition. Height and weight were measured and body mass index was calculated.

### Carotid Artery Stiffness

Testing was performed under quiet resting conditions. The right and left carotid arteries were imaged 3–5 cm proximal to the bulb using B-mode ultrasound with a 9 mHz linear probe (General Electric LOGIQBook). Digital movies were recorded for 10 seconds while the participant was holding his/her breath. Brachial artery blood pressure was taken by an automatic blood pressure machine (Dynamap) immediately before and after the ultrasound images were taken. The digital movies were converted to images (30 per second) and analyzed using semi-automated wall detection software[12]. The same investigator performed all image analyses. Five consecutive pairs of systolic and diastolic diameters were analyzed from each movie. To characterize carotid artery stiffness, the distensibility coefficient (DC) was calculated as DC= (2Δd/D)/Δp where Δp is the pulse pressure, Δd is the difference between systolic and diastolic diameter and D is the end systolic diameter. Distensibility coefficient provides an index of arterial stiffness adjusted for diameter differences seen between men and women.

### Pulse Wave Velocity

PWV was determined two ways (carotid tonometry-femoral pressure and ECG-femoral pressure). For the tonometry method, a force transducer (Grass, Inc) with a 2 mm diameter tip recorded pressure from the carotid artery. For the ECG method, ECG measurements were collected with three electrodes on the chest to measure the 'R' wave. Both methods used waveforms produced by a blood pressure cuff placed proximally on the thigh and inflated to 60 mmHg. The waveforms from the three sites were collected simultaneously at 200 Hz with a Biopac data acquisition system with Acknowledge Software. PWV was calculated as the distance/time. For the tonometry method, the distance between the force transducer on the carotid artery and the cuff on the femoral artery was measured using a tape measure held over the subject to avoid potential body contours. Time was calculated from the foot of the pressure wave at the first point (carotid artery) to the foot of the femoral artery pressure wave over five cardiac cycles. For ECG method, the distance from the approximate location of the heart to the femoral artery was taken using a tape measure. In addition, delay duration of 0.05 seconds was subtracted to account for isovolumetric contraction time.

### Body composition

Body composition was assessed using body mass index and waist to hip ratio. Body mass index was calculated by weight in kilograms divided by height in meters squared. Weight was assessed using a digital weight scale and height was assessed using a stadometer. Waist to hip ratio was taken using a tape measure and measuring waist circumference and dividing it by hip circumference.

### Physical Activity Questionnaire

To assess physical activity for each group, the Baecke Questionnaire of Habitual Physical Activity[11] was used. The questionnaire consists of three sections: work, sport, and non-sport leisure activity. The participants were also asked if they did any yoga, if they answered yes, then they were asked if they did any inversion yoga postures and if so, for how many days a week.

### Nutrition

The participants answered four nutritional questions: 1) Did the participant eat meat and if so how much meat did they eat in a week, 2) Did the participant eat fried foods and if so how often in one week, 3) Did the participant skin their chicken, and 4) How often did the participant eat vegetables in one week. The questions were scored either 0. 0.5, or 1.0 (none or rarely, sometimes, or often, respectively). Only the first two questions were used in the analysis of the results as no differences were seen between groups for the third and fourth questions.

### Statistical analysis

The differences between the three groups with respect to descriptive characteristics were assessed using a one-way ANOVA using Bonferoni comparison (yoga, moderately active, and sedentary by right and left carotid artery stiffness). The influence of physical activity on carotid arterial stiffness was assessed using one-way ANOVA. All models included all three groups and both carotid artery measurements. The outcome variable was central arterial stiffness and data was reported as means ± standard errors. Differences were significant if P < 0.05.

To determine reproducibility, coefficients of variability (CV) were determined for DC, PWV, heart rate and blood pressure. The values for each day consisted of an average of four measurements (two associated with each carotid artery). Bland Altman plots were used to visually inspect the data for any systematic differences in variability. Repeated measures ANOVA was used to determine the significance of difference between visit 1 and visit 2. For comparisons between the left and right carotid artery CV were also determined using an average of four measurements (two each day). Bland Altman plots were used to visually inspect the data for any systematic differences in variability. A paired t-test was used to assess significance differences between the left and right carotid arteries. Coefficients of variation (CV = (S.D./μ)*100) were calculated between the left and right carotid arteries and between the two visits. A CV of less than 10% was used to determine the reproducibility between visit one and visit two and comparison of the left and right sides.

To test the validity of PWV (ECG) and its correlation with PWV (carotid-femoral tonometry) a correlation coefficient was calculated between the two measures. The analysis was done using the averages of both sides and both days.

## Results

The physical characteristics of the subjects are shown in Table 1. There were no significant group differences in age, height, and weight between the three groups. BMI was significantly lower in the yoga compared to the sedentary group (p < 0.05). The total physical activity scores were similar for the yoga and aerobic groups, and both groups had higher total physical activity scores than the sedentary group. When physical activity was separated into work, sports, and leisure activities, the Yoga group had significantly higher activities during sports (2.9 ± 0.5 vs 2.0 ± 0.5, P < 0.01) and leisure (3.4 ± 0.6 vs 2.3 ± 0.6, P < 0.01) compared to the Sedentary group. The Active group had significantly higher activities during sports (3.15 ± 0.3 vs 2.0 ± 0.5, P < 0.01) and leisure (2.6 ± 0.6 vs 2.3 ± 0.6, P < 0.01) compared to the Sedentary group. The Yoga group reported more leisure activity than the Active group (p < 0.001). There were no differences in work activity between any of the groups. The sedentary group had a higher nutritional score compared to the yoga indicating eating more meat and fried foods. No differences in nutritional scores were seen between the aerobic and yoga groups. Blood pressure and heart rate characteristics for the three groups are presented in Table 2. The sedentary group had higher systolic, diastolic, and pulse pressures than the yoga group (p < 0.05). The Sedentary group also had higher pulse pressure than the Active group (P = 0.011).

**Table 1:** Selected subject characteristics

	Age (yrs)	Female/Male (Total)	Height (m)	Weight (kg)	BMI (kg/m^2^)	W-H ratio	Food Score	Physical Activity Score
Aerobic Group	51.9 ± 7.2	4/6 = 10	1.69 ± 0.10	70 ± 13	24.2 ± 2.1	0.82 ± 0.11	0.53 ± 0.28 *	8.04 ± 0.82 #
Yoga Group	48.1 ± 5.8	5/3 = 8	1.72 ± 0.08	68 ± 10	22.9 ± 2.9 *	0.80 ± 0.05 *	0.41 ± 0.27 #	8.56 ± 1.37 #
Sedentary group	51.0 ± 8.2	3/5 = 8	1.77 ± 0.13	83 ± 19	26.5 ± 2.9	0.87 ± 0.06	0.81 ± 0.26	6.29 ± 0.94

W-H is the waist to hip ratio. Food score is from 0–1. *p < 0.05 versus Sedentary group. # p < 0.01 versus Sedentary group. Values are means ± SD.

**Table 2:** Subject blood pressure characteristics

	SBP (mmHg)	DBP (mmHg)	PP (mmHg)	HR (beats/min)
Aerobic	115 ± 12	71 ± 6.9	43 ± 6.8*	60 ± 11
Yoga	108 ± 8.9#	65 ± 6.2*	43 ± 4.2*	63 ± 9.2
Sedentary	124 ± 7.6	72 ± 6.6	52 ± 5.6	67 ± 9.8

PP is pulse pressure (systolic – diastolic). *p < 0.05 sedentary versus Yoga and Aerobic groups. #p < 0.01 Aerobic versus yoga group. Values are means ± SD.

**Figure 1:**
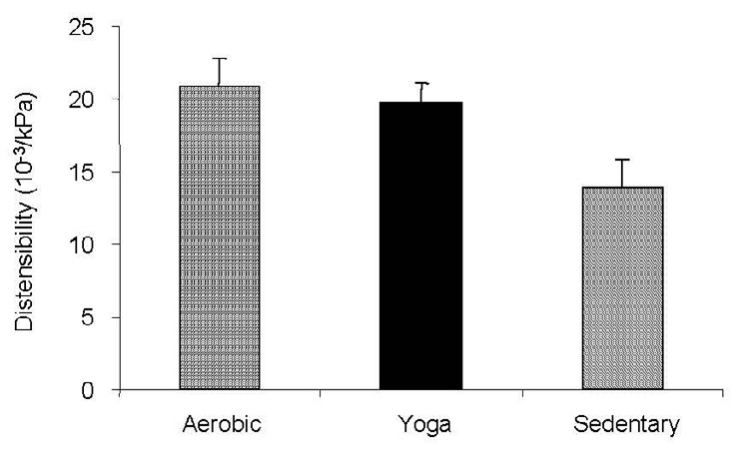
Distensibility values for the carotid artery for all subjects. The sedentary group had significantly lower distensibility than the aerobic and the yoga groups (P < 0.001). Data reported as Means ± S.D.

Mean distensibility for the aerobic, yoga, and sedentary groups are shown in Figure 1. Carotid artery stiffness measured by distensibility was not a statistically significantly different between the aerobic group and the yoga group (p value was 0.26 and the effect size was 0.04). A significant difference in distensibility was seen between the Sedentary group and the Yoga and Aerobic groups (p < 0.001 with an effect size of 0.84).

**Figure 2:**
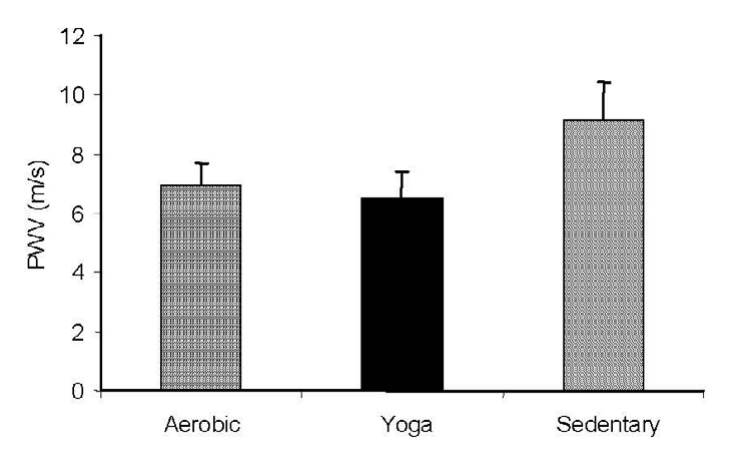
Pulse wave velocity values for all subjects. The sedentary group had significantly higher pulse wave velocity than the aerobic and the yoga groups (P < 0.001). Data reported as Means ± S.D.

Mean PWV between the aerobic, yoga, and sedentary groups are shown in Figure 2. Central stiffness measured by PWV was not significantly different between the aerobic group and the yoga group (p = 0.21 with an effect size of 0.08). There was a significant difference seen between the Sedentary group and the Yoga and Aerobic groups (p < 0.001 with and effect size of 0.81).

**Figure 3:**
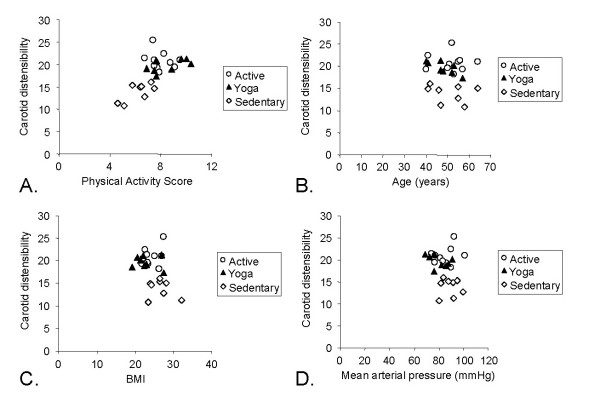
The relationship between carotid artery distensibility and A) physical activity score, B) Age, C) BMI, and D) mean arterial pressure. Note that yoga and aerobically active groups were similar while the sedentary group had lower distensibility values. With all groups combined the correlation between distensibility and physical activity was statistically significant (R^2 ^= 0.43 with p < 0.01).

Correlations between carotid distensibility and physical activity, age, BMI, and MAP are shown in Figure 3. Using a regression model with age, BMI, MAP, and total physical activity, only physical activity was significant (R^2 ^for the model was 0.477, P = 0.009: physical activity t = 3.41, p = 0.003). PWV measured using the tonometry method showed a similar relationship with physical activity (R^2 ^for the model was 0.658, P < 0.001: physical activity t = -5.28, p < 0.001), although BMI was also a significant predictor (t = 2.15, p = 0.044).

Comparison of measurements from visits one and two are seen in Table 3. All of the measurements taken showed good reproducibility from visit one to visit two. (CV<10% for all measurements). The strongest reliability was seen with distensibility and PWV as the coefficients of variation were less than 3%.

**Table 3:** Comparison of measurements Day 1 and Day 2

	HR (beats/min)	SBP (mmHg)	DBP (mmHg)	PP (mmHg)	Diameter Change (mm)	DC (kPa)	PWV ECG (m/s)	PWV (carotid-femoral) (m/s)
**Visit 1**	61 ± 10	113 ± 25	70 ± 16	45 ± 11	0.61 ± 0.11	18.3 ± 3.5	7.72 ± 1.85	7.56 ± 1.49
**Visit 2**	61 ± 11	112 ± 25	70 ± 16	43 ± 11	0.60 ± 0.20	18.4 ± 3.4	7.73 ± 1.86	7.57 ± 1.48
**Correlation**	0.98	0.96	0.92	0.84	0.82	0.98	0.99	0.98
**CV**	2.7%	6.2%	3.3%	6.6%	7%	2.5%	2.3%	1.8%

PP is pulse pressure (systolic – diastolic). Diameter change is diastolic – systolic diameter. DC is distensibility. No significant differences between the left and right side were found for all measurements. Values are means ± SD.

**Figure 4:**
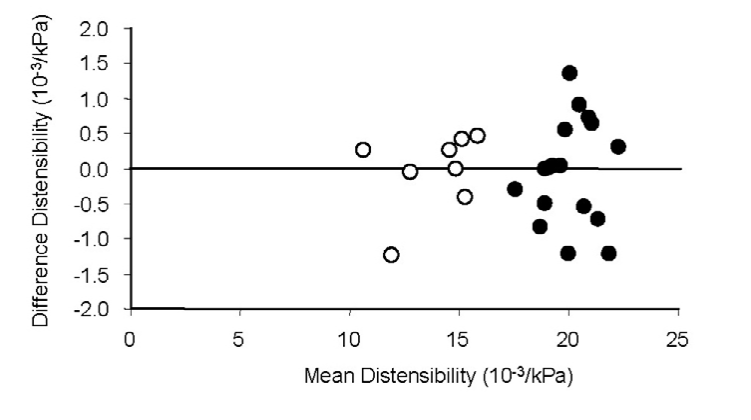
The Bland Altman plot of left and right carotid artery distensibility. The sedentary subjects are show in open symbols and the other two groups in solid symbols. The maximum range of the scale (+0.02, -0.02) indicates the +/- 2 SD range.

Comparisons of measurements from the left and right carotid arteries are seen in Table 4. All measurements had a CV < 10%. The highest agreement of the left side to the right side were between with distensibility (Figure 4) and PWV, which all had CV of <3%. The lowest agreement was seen in pulse pressure and diameter change which both had a CV of greater than 4%.

**Table 4:** Comparison of measurements left versus right side

	Heart Rate (beats/min)	SBP (mmHg)	DBP (mmHg)	PP (mmHg)	Diameter Change (mm)	DC (kPa)	PWV ECG (m/s)	PWV (carotid-femoral) (m/s)
**Left**	63 ± 10	116 ± 12	70 ± 9.0	46 ± 6.7	0.61 ± 0.11	18.3 ± 3.4	7.69 ± 1.89	7.67 ± 1.73
**Right**	64 ± 11	117 ± 14	71 ± 8.6	46 ± 7.5	0.60 ± 0.11	18.4 ± 3.5	7.76 ± 1.82	7.72 ± 1.64
**Correlation**	0.96	0.95	0.93	0.85	0.82	0.98	0.97	0.98
**CV**	3.4%	6.2%	4.0%	6.8%	6.9%	2.2%	1.8%	1.7%

PP is pulse pressure (systolic – diastolic). Diameter change is diastolic – systolic diameter. DC is distensibility. No significant differences between the left and right side were found for all measurements. Values are means ± SD.

There was a very strong correlation between the tonometry and ECG methods of measuring PWV. The correlation coefficient calculated was to be 0.93 and the prediction equation had a slope near one with an intercept of -0.59.

## Discussion

This study found that physical activity had the greatest influence on arterial stiffness in middle-aged men and women. This is consistent with previous studies that have shown that high physical activity is associated with reduced arterial stiffness [13-15]. Tanaka et al.[2] found that arterial compliance in trained middle aged and older groups was 20 to 35% higher than in their sedentary groups, consistent with what we found for our aerobic and yoga groups (32% higher than our sedentary group). Kupari et al.[16] found a significant correlation between arterial distensibility and self-reported physical activity (R^2 ^= 0.45), similar to our correlations with arterial compliance (R^2 ^= 0.52 distensibility and 0.50 PWV).

Part of the rationale for this study was the observation that recreational practice of Yoga can be associated with performing inversion postures. Inversion postures such as those performed during Hatha Yoga, serve to increase blood flow but also blood pressure to the head. Increased blood pressure resulting from both acute [8] and chronic resistance [9] training has been associated with increased carotid artery stiffness. However, we did not find evidence of increased arterial stiffness in our yoga group. A likely explanation for this is that the high pressure stimulus in our study was not enough to produce arterial stiffening. The resistance exercise studies that have evaluated carotid artery stiffness used longer durations of resistance exercise than time our subjects spent in inversion postures. Future studies will be needed to more carefully classify the pressure stimulus associated with practicing yoga with inversion postures.

As expected, the sedentary participants had significantly lower levels of self-reported physical activity than the aerobic group. However, the yoga group was more active than expected and had physical activity scores similar to those of the aerobic group. Because of this, we were unable to determine an independent affect of yoga on arterial stiffness. The yoga participants performed some form of inversion postures (head stand or shoulder stand), a practice that has been shown to increase blood flow to the carotid artery during the posture[17]. While the effect of yoga with inversion posture on arterial stiffness has not been studied before, several studies have found that people who practice yoga and inversion postures had a lower resting heart rates and systolic and diastolic blood pressures compared with people who are sedentary[3,5,6]. A potential limitation to these studies are that they did not report the physical activity levels of the subjects who performed yoga[3,6,7]. Our study suggests that physical activity levels in addition to the practice of yoga needs to be measured in order to interpret the influence of yoga on cardiovascular risk factors for disease.

The yoga and aerobic groups reported to eating less meat and less fatty foods than the sedentary group, and they had significantly lower BMI, waist to hip ratio, and systolic blood pressure. Previous studies have shown that diet can influence cardiovascular function[18]. A potential limitation to our study was that our nutritional assessments were limited and can't provide a definitive conclusion on the effects of diet on artery stiffness. Our results do suggest that future studies should incorporate more detailed methods of measuring nutrition when evaluating the effects of yoga and physical activity on arterial stiffness.

Several studies have shown a strong correlation between age and arterial stiffness[2,14]. Our study used middle aged participants (40–65 yrs) because this is the age range where stiffness starts to occur in the arteries and where different physical activity levels tend to have the greatest impact on arterial stiffness[2]. We did not find a significant effect of age on either carotid distensibility or PWV in our subjects, perhaps due to the large effect of physical activity and the relatively small age range of 24 years. Consistent with our findings Tanaka et al.[2] did not find significant age effects on arterial compliance within their group of middle-aged participants.

Our study found a very good agreement (R^2 ^= 0.91) between measurements of PWV using two different approaches for determining the start of the pressure wave at the heart, carotid tonometry and ECG with a constant delay. Several studies have compared different ways of measuring PWV but none have focused on ECG and carotid tonometry[19]. The PWV values obtained from this study (6.5–6.9 m/s for active and 9.0 m/s for sedentary) were similar to the values obtained by McEniery et al.[20] study for healthy adults 40–70 years of age (6 to 9 m/s). Carotid tonometry is the gold standard when measuring PWV but ECG is easier to use and less tedious. One possible limitation of ECG method is that it assumes a constant isovolumetric contraction time of 0.05 seconds for all subjects. For this reason, ECG may not be an accurate measure of PWV. However, the limitation to carotid tonometry is that it requires a skilled operator to hold the tonometry device. Our study showed that PWV measured with ECG correlated well with PWV measured using carotid tonometry.

We found good day-to-day reproducibility for arterial stiffness in our study. The coefficient of variation for distensibility and PWV were between 2–3% of the mean value. These values are similar to those of Liang et al.[10] for distensibility and PWV over two visits(CV = 10% and 3.2%, respectively)[10]. We also found good reliability comparing left and right arterial stiffness (CV = 2.2 and 2.7 for distensibility and PWV). Most research studies report measurements of carotid artery stiffness from only one (typically the right) side. While arteriosclerosis is known to progress in an asymmetrical manner, we found no evidence that both arteries need to be assessed in the middle-aged healthy adults. This does not mean that studies on older or less healthy adults should not test both arteries.

## Limitations

A potential limitation of our study was that the participants were not asked if they performed high intensity resistance training. Subjects who perform regular resistance training may have greater central arterial stiffness[21]. However, it maybe that very high intensity resistance exercise is needed to increase arterial stiffness, which is unlikely in our population of recreationally active adults. We also did not record the intensity of the aerobic exercise performed by our yoga and aerobic activity groups, we only asked how many days a week and for long they exercise. Previous studies have shown that intensity of aerobic exercise may influence arterial stiffness[13]. Another limitation is that the yoga participants were not asked the duration of the inversion postures they performed. Future studies will be needed to tease out the potential effects of inversion yoga on arterial stiffness.

The yoga and aerobic groups reported to eating less meat and less fatty foods than the sedentary group, and they had significantly lower BMI, waist to hip ratio, and systolic blood pressure. Previous studies have shown that diet can influence cardiovascular function[18]. A potential limitation to our study was that our nutritional assessments were limited and can't provide a definitive conclusion on the effects of diet on artery stiffness. Our results do suggest that future studies should incorporate more detailed methods of measuring nutrition when evaluating the effects of yoga and physical activity on arterial stiffness.

## Conclusion

In this study of middle-age men and women, physical activity had the greatest influence on arterial stiffness. Yoga participants had significantly lower arterial stiffness than sedentary participants, but they also reported aerobic activities similar to our aerobic group. Futures studies on the influence of yoga will need to control for physical activity levels, as well as nutritional habits. This study found a high level of agreement between measurements of carotid distensibility and two different methods of measuring PWV, suggesting that any of the measures could be used to evaluate central arterial stiffness. This study also found that in the age group tested, there was no evidence justifying the need to measure distensibility in both the left and right carotid arteries.

## Competing interests

The author(s) declare that they have no competing interests.

## Authors' contributions

CMD conducted the experiments, performed initial data analysis, and drafted the manuscript. MEC participated in the preparation of the study design and in writing the manuscript. KKM conceived of the study and participated in its design and coordination, and in writing the manuscript. All authors read and approved the final manuscript.
